# Spider venom potency exhibits phylogenetic prey specificity but does not trade-off with body size or silk use in prey capture

**DOI:** 10.1098/rsbl.2025.0133

**Published:** 2025-05-21

**Authors:** Keith Lyons, M. M. Dugon, Kevin Healy

**Affiliations:** ^1^ Zoology Department, University of Galway, Galway, Ireland

**Keywords:** spider, venom potency, LD_50_, prey-specific, venom yield, phylogenetic comparative analyses

## Abstract

Spiders employ a diverse range of predator traits, including potent venoms, complex silk-hunting strategies and mechanical strength coupled with larger body sizes to capture prey. This trait diversity, along with the quantifiable nature of venom potency, makes spiders an excellent group to study evolutionary trade-offs. Yet, comparative approaches have been historically confounded by the use of atypical prey models to measure venom potency. Here, we account for such confounding issues by incorporating the phylogenetic similarity between a spider’s diet and the species used to measure its venom potency. Using a phylogenetic comparative analysis of 75 spider species to test how diet, silk use in prey capture and body size drive venom yield and potency (LD_50_), we show that spider venoms are generally more potent against models more closely related to their natural prey, reflecting prey-specific patterns. Despite predictions, we find no trade-offs among body size, silk use and venom potency. We find that venom yield scales sublinearly with size, reflecting the 0.75 allometric scaling predicted by metabolic theory, suggesting that venom is metabolically expensive in spiders. Our approach demonstrates how contemporary comparative approaches can be applied to historic venom potency measures to test fundamental evolutionary patterns in predator traits.

## 1. Introduction

How predators capture prey varies greatly from mechanically overpowering prey [1,2] to biochemical strategies, including venom [3,4]. Typically, predators employ a suite of traits that work in concert to incapacitate prey, such as jaws and limbs [2,5], which, in turn, may cause evolutionary trade-offs, with investment in one set of traits at the expense of another. For example, scorpions with larger body sizes and pincers were found to have lower venom potencies [6]. While knowledge of such trade-offs is growing, suitable examples for exploring potential trade-offs between multiple predator traits are limited [7]. Spiders, however, provide fertile ground to test such trade-offs.

Spiders employ a suite of contrasting predator traits to capture prey, including potent venoms, specialized silk-hunting strategies and mechanical strength, which is typically coupled with larger body sizes [8–10]. This leads to multiple potential evolutionary trade-offs. For example, non-silk hunters may have more potent venoms than species that use both venom and silk, as both traits likely incur metabolic costs [9,11,12]. Other trade-offs are likely associated with body size. For instance, relatively large spiders, such as the Theraphosidae, may rely more on mechanical strength and large chelicerae (fangs) to dispatch prey, resulting in reduced selection for potent venoms [10,11,13]. Such trade-offs may also be reflected through allometric scaling [14–16]. For example, Paillard & Arbuckle [17] found evidence of negative allometry in web size relative to spider size, suggesting that larger spiders build proportionally smaller webs, likely to reduce metabolic costs associated with silk production [12]. Similarly, while larger species generally have higher venom yields [14], they may have lower yields relative to their size due to metabolic costs associated with venom production [11,18].

While spiders offer an ideal group for testing evolutionary trade-offs, comparative approaches have historically been limited by the prey-specific nature of venoms, with potency measures, such as median lethal dose (LD_50_) [19], often incorporating murine models [20–22], despite the fact that spiders predominantly prey on arthropods [23,24] and occasionally on annelids, gastropods and small non-murine vertebrates [10,24,25]. The prey-specific hypothesis postulates that predator venoms are most effective against their natural prey [26], with the degree of prey specialization in spiders ranging from class to genus level, with one instance at species level [26–28]. Prey-specific venoms (family-genus level) have been observed in some prey-specialized spiders [29–31] and were found to be the general pattern in snakes [18]. However, whether this is a general pattern in spiders or if this pattern is observed at other degrees of prey specificity, such as class-order level, is poorly understood.

**Figure 1.**
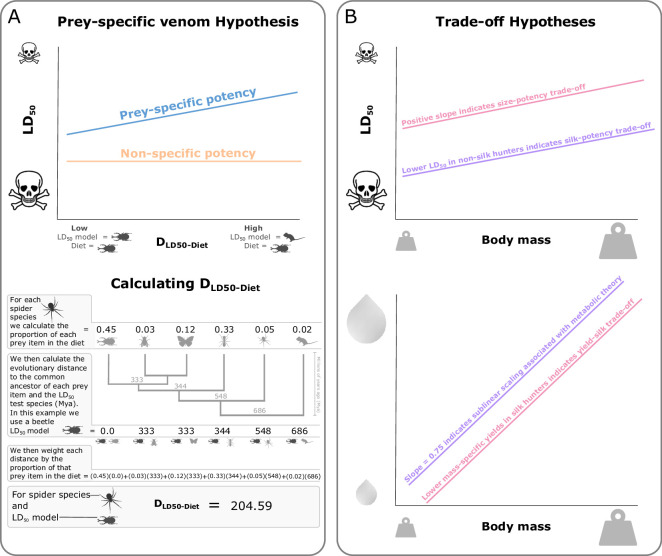
Summary of hypotheses. (A) Phylogenetic prey-specific venoms (blue) are predicted to produce lower potencies (higher LD_50_) in injected model species that are more evolutionarily distant from natural prey, as measured using *D*_LD50-Diet_. *D*_LD50-Diet_ is the mean phylogenetic distance between the injected model species and the natural prey species, measured in hundreds of millions of years (Hmya), and is weighted by the proportion of each prey item in the diet. An example of calculating *D*_LD50-Diet_ is provided. (B) In the presence of a trade-off between venom potency and body mass, we predict a positive relationship between LD_50_ and body mass (pink, top), converted from mean spider body length. In the presence of a trade-off between venom potency and silk use in prey capture, we predict non-silk hunters to produce stronger potencies (lower LD_50_) in general when compared to silk hunters (purple, top). In the presence of trade-offs between venom yield and body mass, we predict yield to scale sublinearly with mass via the 0.75 allometric scaling exponent predicted by metabolic theory [32] (purple, bottom) and for silk hunters to produce lower lyophilized venom yields than non-silk hunters (pink, bottom).

One approach that both tests for patterns of prey specificity in venoms and accounts for test model variation is to measure the distance between a spider’s natural diet and the model species its venom was tested on, referred to as *D*_LD50-Diet_ (figure 1A) [18,33]. By quantifying the phylogenetic prey specificity (*D*_LD50-Diet_) of spider venoms, we can test if venoms become less potent when tested on species that are more evolutionarily distant from natural prey, which would indicate a general pattern of prey specificity (figure 1A). The phylogenetic prey-specificity approach (*D*_LD50-Diet_) has already demonstrated prey specificity as a general pattern in snakes [18], but it has yet to be applied to spiders. As this approach accounts for variation associated with the confounding factor of prey specificity, it also provides a comparative method to test for trade-offs between contrasting predator traits.

Using a phylogenetic comparative approach, we test the phylogenetic prey specificity of spider venoms. Since phylogenetic prey-specificity may be particularly associated with narrower, more specialized diets, as found in snakes [33], we also test whether it is mediated by diet taxonomic class richness (diet breadth). Within this comparative framework, we also test for trade-offs between predatory traits, predicting (i) that larger species produce lower venom potencies (higher LD_50_) than smaller species, as larger species may rely more on mechanical strength and chelicerae to incapacitate prey [6,10,13]; (ii) that silk hunters produce lower venom potencies (higher LD_50_) than non-silk hunters as they use silk to help immobilize their prey, potentially reducing selection for potent venoms; (iii) that silk hunters produce lower venom yields to reduce metabolic costs [11,12]. Finally, (iv) we predict an exponent of 0.75 for the allometric scaling between spider venom yield and body size, reflecting predictions from metabolic theory [18,34], where production rates of venom are associated with general metabolic rates and how they scale with body size (figure 1).

## 2. Material and methods

### (a) Data collection

To test our hypotheses, we collated data on venom potency, body size, silk use in prey capture, venom yield, LD_50_ model species and natural diet from The World Spider Trait database [35] and other literature sources (electronic supplementary material, S1). For venom potency, we used LD_50_ [19], administered via intraperitoneal (IP), intravenous (IV) or subcutaneous (SC) routes for vertebrate models or via thorax/cephalothorax or abdomen for invertebrate models. We only included LD_50_ values reported as milligrams of lyophilized venom per kilogram of test subject (mg kg^−1^) or convertible to mg kg^−1^. Non-lyophilized LD_50_ measures were only included if values were in mg kg^−1^ or convertible to mg kg^−1^, based on protein content estimation methods [36,37].

For body size, as prosoma lengths were not available for most species, we used mean total spider body length (mm) and converted it to body mass (g) using the power law Mass = Length^2.6^ based on a generalized linear model (electronic supplementary material, S5 and S6). Silk use in prey capture was recorded under two categories, denoting if a species utilized silk to directly capture prey or not. Species that use silk for auxiliary, but not direct, functions related to prey capture were defined as not utilizing silk in prey capture. For example, we defined many Theraphosidae that lay silk outside burrows, where it functions primarily in prey detection but not in prey capture, as not utilizing silk for prey capture [38]. For venom yields (milligrams per spider), we recorded whether the extraction method was ‘electrical stimulation’, ‘direct stimulation’ of agitated spiders to collect venom via a capillary tube or bite into a cup capped with Parafilm or ‘venom gland extraction’, to control for potential extraction effects [39] and whether venom was lyophilized (dried).

Dietary data were collated from the literature using studies with quantitative estimates when available, recorded as the percentage proportion of each prey group in the diet (figure 1A). Following how dietary items were most frequently reported in the literature, we recorded diet at class level for Malacostraca (woodlice), Diplopoda, Chilopoda, Gastropoda, Mammalia, Amphibia, Squamata and Aves, and at superclass level for Osteichthyes (bony fish). The remaining prey were recorded at the order level for class Insecta (21 orders), Arachnida (six orders) and Opisthopora (earthworms). When quantitative data were unavailable, we used qualitative descriptions to score diet contributions. When the proportion of a prey item in a species diet was described as a major component, we scored it as accounting for 60% of the diet, common as 20%, uncommon as 5% and rare as 1%. While keeping these scores in proportion, we then adjusted them, so the sum of the proportions equalled 100% (table 1, electronic supplementary material, S6). For species with limited or no diet data but reported as having similar diets to a relative at the genus level or family level for specific Theraphosidae, we inferred the diet (electronic supplementary material, S1). Eleven species had insufficient diet data or any closely related species with similar diets to infer natural diet from and so were excluded from the main LD_50_ model. To test the effect of diet class richness on venom potency, we recorded how many prey classes were included in each species’ diet with a max of 12 classes.

**Table 1.** Example demonstrating how qualitative data was scored and converted to quantitative data. We present *Amaurobius similis* as an example with both qualitative and quantitative reports. The qualitative data was determined based on the literature descriptions [40–42]. The quantitative data equals 97% as 3% of items were classified as ‘other’ for that particular study [24]. The ‘weighted combined estimate’ is the result of taking the sum of all unweighted proportions given to the qualitative data and the quantitative data (1.45 in this case) and dividing each group’s proportion in the spider’s diet by the sum of all proportions, so the resulting sum of all weighted proportions equals 100% ± 1%. For example, for Coleoptera (first group): 0.21/1.45 = 0.144 (14.4%). A step-by-step description is provided in electronic supplementary material, S6.

taxonomic prey groups reported in *A. similis* diet								
	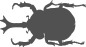	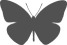	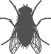	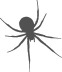	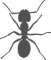	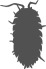	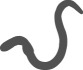	proportion of diet (total =100%)
reported qualitative	common	common	major	uncommon	rare	common	rare	_
reported quantitative	21%	_	75%	_	1%	_	_	97%
unweighted combined estimate	0.21 (21%)	0.21 (21%)	0.75 (75%)	0.05 (5%)	0.01 (1%)	0.21 (21%)	0.01 (1%)	1.45 (145%)
weighted combined estimate	0.144 (−14.40%)	0.144 (−14.40%)	0.517 (−51.70%)	0.034 (−3.40%)	0.007 (−0.70%)	0.144 (−14.40%)	0.007 (−0.70%)	0.997 (−99.70%)

To quantify phylogenetic prey-specific patterns in venom potency, we used mean phylogenetic distance, measured as the weighted divergence time (Hmya), between the test model and natural prey species [18,33] (figure 1*a*). Phylogenetic distances were calculated using TimeTree [43] (electronic supplementary material, S1). To create a phylogeny of the species in our dataset, we used the phylogeny from Wolff *et al*. [44] as our backbone phylogeny. Species in our dataset not included in Wolff’s phylogeny were added to the phylogeny based on evidence from the literature [45–55] (electronic supplementary material, S4 and S6). To test if the silk use trait is constrained to a single phylogenetic clade, we also performed ancestral character estimation using maximum likelihood for discrete characters [56].

### (b) Set-up of Markov chain Monte Carlo generalized linear mixed models

To test our hypotheses, we fitted Bayesian phylogenetic mixed models, using the Markov chain Monte Carlo generalized linear mixed model (MCMCglmm) package [57] in R v. 4.4.2 [58], which allows for the inclusion of variance terms to account for multiple observations per species and the inclusion of a phylogenetic term [57]. We controlled for pseudoreplication due to shared ancestry between species, through the ‘animal’ term, which uses a distance matrix of the phylogenetic distance between species to control for the expected similarity in factor values. We then calculated the relative variance attributable to the animal term as *h*^2^, which can be interpreted similarly to the phylogenetic lambda value [59]. To include multiple LD_50_ measures for each species in our analysis, we used a random term for species, similar to previous comparative models of venom variation [18,33]. As the MCMCglmm is a Bayesian approach that requires specifying priors, we fit all models using standard flat non-informative priors, which assume no prior expectations for the estimated values across the model. We used a burn-in of 40 000 and a thinning of 100 over 2 400 000 iterations to ensure that the sample sizes exceeded 1000 for all parameter estimates. We tested for convergence using the Gelman–Rubin statistic over three separate chains [57]. Significance of an estimate is determined when the 95% credibility interval (CI) does not cross zero [57].

We ran two main models, the first comparing venom potency with body mass, silk use, *D*_LD50-Diet_, diet class richness and injection method, and the second comparing venom yield with body mass and silk use. We included log_10_ of LD_50_ and log_10_ of venom yield as the response variables, with log_10_ of body mass, *D*_LD50-Diet_, diet class richness and an interaction term between *D*_LD50-Diet_ and diet class richness as the independent variables. An interaction term between *D*_LD50-Diet_ and diet taxonomic richness was included to determine if the effect of *D*_LD50-Diet_ on venom potency is mediated by diet class richness [33]. Silk use was included as a fixed factor. We controlled for injection route effects in the LD_50_ model by including it as a fixed factor.

Additionally, we ran four supplementary models (electronic supplementary material, S5). The first model tested whether the presence of vertebrate prey in a spider’s diet affected potency by including a fixed factor for the presence/absence of vertebrates reported in the diet. The second model replicated the main LD_50_ model but without any diet factors to determine how body mass, silk use and venom injection method interact with LD_50_ for all 75 spider species, including previous exclusions. The third and fourth models tested the effects of different venom extraction methods on venom potency (third model) and yield (fourth model), respectively, by including fixed terms for the venom extraction method used (electrical stimulation, direct stimulation or venom gland extraction) and if protein content was determined through lyophilization (dried) or estimated (not dried).

## 3. Results

**Figure 2.**
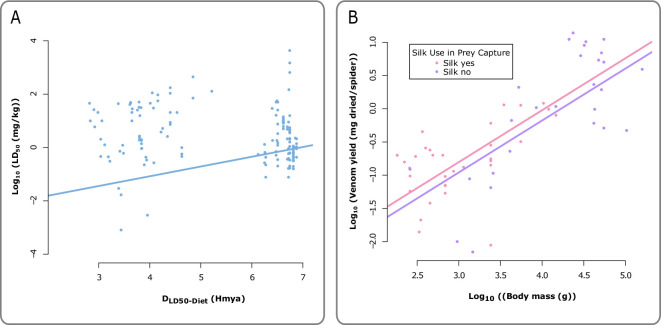
(A) The fitted line highlights the significant positive relationship between *D*_LD50-Diet_ (Hmya) and log_10_ LD_50_ (mg kg^−1^) for 153 measures of LD_50_ across 64 species venoms. (B) The fitted lines show the significant positive relationship between log_10_ body mass (G) and log_10_ venom yield (mg/spider) for 60 venom yield measures across 47 species and that silk hunters (pink) do not produce noticeably higher yields than non-silk hunters (purple).

We found that phylogenetic prey-specificity of venom potency was a general pattern in spiders, with lower log_10_ (LD_50_) values observed when LD_50_ was measured on models more closely related to natural prey at class-order level (*β* (slope) = 0.4, lower 95% CI = 0.05, higher 95% CI = 0.76; figure 2A; electronic supplementary material, S5). We observed significantly higher log_10_ (LD_50_) values when venom was injected via the thorax/cephalothorax route (*β* (intercept difference) = 2.01, lower 95% CI = 1.35, higher 95% CI = 2.67; electronic supplementary material, S5) and abdomen route (*β* (intercept difference) = 1.01, lower 95% CI = 0.22, higher 95% CI = 1.83; electronic supplementary material, S5) when compared to the IP route, but no significant effects were observed with other routes. We found no support for a trade-off between body size and venom potency, and no support for a negative relationship between silk use and venom potency (electronic supplementary material, S5), supporting the absence of a trade-off between venom potency and silk use. We also found no support for a mediating role for diet class richness on venom potency (electronic supplementary material, S5).

The sensitivity analyses supported the findings of the main LD_50_ model, with phylogenetic prey-specificity found to be significant regardless of vertebrate presence/absence in a species’ diet and a lack of support for trade-offs when tested in the full 75-species model without diet-related factors (electronic supplementary material, S5). There was also no support for an effect relating to a method of venom extraction on potency or preparation (electronic supplementary material, S5). Across all LD_50_ models (main model 1, models electronic supplementary material, S2–S4), the phylogenetic signal ranged from 0.003 to 0.005.

For venom yield, we observed a significant positive relationship between log_10_ body mass (g) and log_10_ venom yield (milligrams per spider) (figure 2B; electronic supplementary material, S5) with the slope corresponding to a scaling exponent of 0.73 (*β* (slope) = 0.73, lower 95% CI = 0.34, higher 95% CI = 1.07; electronic supplementary material, S5), indicating a sublinear increase in venom yield with body mass. Conversely, we observed no significant relationship between log_10_ venom yield (mg/spider) and silk use. There was also no support for an effect relating to a method of venom extraction on potency or preparation for yield (electronic supplementary material, S5). The phylogenetic signal for both venom yield models (main model 2, model S5, electronic supplementary material) ranged from 0.86 to 0.89, while the ancestral character estimation demonstrates that the silk use trait is not constrained to one phylogenetic subclade (electronic supplementary material, S5).

## 4. Discussion

Across the diversity of species in our dataset, we found spider venoms exhibit a general pattern of phylogenetic prey-specificity, following the predicted trend that venoms are more effective against prey models evolutionarily closer to natural prey, reflecting similar comparative analyses performed with snakes [18,33]. Our results support evidence of prey-specific venoms from studies on prey-specialized spiders [29–31] and from studies identifying prey-specific toxins in spider venoms [60–64]. As prey-specific patterns are reported in multiple venomous predator groups, including spiders [29–31], snakes [18,33] and cone snails [65], it is likely that prey specificity is a general trend across venomous predators. While we observed a general prey-specific pattern, in contrast to other venomous groups [30,31,33], there was no support for a mediating role of diet breadth in this pattern. This likely reflects the hyper-generalized diets of most spiders, as indicated by the median diet class richness in our dataset of four classes. However, while studies focusing on prey-specialized spiders demonstrate strong prey specificity in their venoms (family genus level) [26], our results suggest that such prey specificity is not restricted to such trophic specialists.

Despite predictions and contrary to observations by Quistad *et al*. [66], we found no support for a trade-off between spider body size and LD_50_. The lack of a size-potency trade-off may highlight that spiders, regardless of their size, generally rely on venom to subdue prey in some capacity. In particular, some spider families may have evolved potent venoms and relatively large body sizes to work complementarily with one another in response to potentially dangerous vertebrate prey [10,24], such as in *Phoneutria* sp. that produce significantly lower LD_50_ values in vertebrates compared to arthropods and rely on their large chelicerae and elongated legs to restrain prey while delivering their venomous bite [24,67].

We also find no support for a trade-off between silk use in prey capture and venom potency. While some previous studies show support for a trade-off between venom and silk in prey-specialized Gnaphosidae spiders [9], our results align with previous studies that observed a lack of trade-offs in other Gnaphosidae [68] and Tetragnathidae spiders [69]. These results indicate that while hunting strategies that rely predominantly on a single trait exist [9,70], the majority of species using silk in prey capture use both silk and venom complementarily to capture prey, with other factors, such as environment type and prey risk, likely to be the primary drivers of the evolution of silk-hunting behaviours and venom potency.

As expected, we observed a significant relationship between spider venom yield and size, with venom yield scaling sublinearly with size via an exponent of 0.73, closely reflecting the 0.75 allometric scaling predicted from metabolic theory. This suggests that venom production is potentially constrained by processes relating to metabolic scaling [32], reflecting a similar pattern observed in snakes [18] and previous studies that suggest venom is metabolically expensive [11,15,71,72]. While larger species still generally produce higher venom yields, as demonstrated here and by Herzig *et al*. [73], our results suggest that relative to their own size, larger species typically produce lower lyophilized venom yields than smaller species.

While the historical availability of spider LD_50_ measures and its associated broad taxonomic coverage allows for the use of spider LD_50_ measures in large comparative analyses such as ours, its insights into various functional aspects of spider venoms are likely limited. This is because spider venoms are expected to be under selection to primarily incapacitate prey [74–76], with lethality likely being a secondary effect. Therefore, incorporating other measures of venom potency in future analyses, such as median effective dose (ED_50_) [39,77] as well as more prey-centric traits such as prey size, will likely aid in identifying the fundamental drivers of spider venom potency. Furthermore, little is known regarding the role of defence in spider venom potency. For example, notably low LD_50_ values were observed in male *Atrax* spp. venoms when tested in murine models, despite *Atrax* spp. not naturally preying upon mammals [78], indicating that roles other than predation can seriously influence spider venom potency. Phylogenetic comparative approaches, such as those described here, can aid in determining whether such factors play a general or more idiosyncratic role in the evolution of not only spider venoms but venoms in general.

## Data Availability

All data and code are included in electronic supplementary material S1–S7, which includes both datasets (S1, S2), code to reproduce the results (S3) and phylogeny (S4), a document with all MCMCglmm model results and the Ancestral Character Estimation for Silk Use in Prey Capture (S5) and a document detailing the methodology in greater detail, along with data descriptions (S6). A list of all references from both datasets that are not cited directly in this article is also available (S7). The supplementary material is available from the Dryad Digital Repository [79]. The spider body size, venom yield and venom potency (LD_50_) data can also be found in the World Spider Trait database (https://doi.org/10.57758/4A6E-QJ04). Supplementary material available online [80].
